# Supplementary material for: Visualizing protein breathing motions associated with aromatic ring flipping
Source: Nature. 2022 Feb 16;602(7898):695–700. doi: 10.1038/s41586-022-04417-6 (PMC8866124; doi:10.1038/s41586-022-04417-6)
Supplement: Supplementary file 1 — This file contains the Supplementary Discussion (sections 1–3), Supplementary Fig. 1 and Supplementary Table 1. [file 41586_2022_4417_MOESM1_ESM.pdf]

nature portfolio

https://doi.org/10.1038/s41586-022-04417-6

# Supplementary information

# Visualizing protein breathing motions associated with aromatic ring flipping

In the format provided by the authors and unedited

# Supplementary Information

# Visualizing protein breathing motions associated with aromatic ring flipping

Laura Mariño Pérez, Francesco S. Ielasi, Luiza M. Bessa, Damien Maurin,

Jaka Kragelj, Martin Blackledge, Nicola Salvi, Guillaume Bouvignies,

Andrés Palencia*, Malene Ringkjøbing Jensen*

*Correspondence to: andres.palencia@inserm.fr or malene.ringkjobing-jensen@ibs.fr

**This file includes:**

Supplementary Discussion (p. 2-4)

Supplementary Figure 1 (p. 5)

Supplementary Table 1 (p. 6-7)

## Supplementary Discussion

### 1. Lipari-Szabo model-free analysis of $^{15}N$ relaxation data

A Lipari-Szabo model-free analysis was carried out using the program Tensor2 of the data acquired at 25°C. The diffusion tensor was determined on the basis of residues with no significant exchange contributions (as judged from the ratio of $^{15}N$ $R_2$ and $R_1$) and for which the experimental heteronuclear NOEs were above 0.7 (residues 492-495, 502-516 and 528-538). Analysis of the data according to an isotropic model for the diffusion tensor gives a rotational correlation time of 8.5 nanoseconds consistent with a dimeric protein. A complete model free analysis of the data shows that the diffusion tensor is axially symmetric with the main axis effectively aligned (2° difference) along the unique axis of the inertia tensor calculated from the structure of the dimer of JIP1-SH3. To monitor the influence of protein concentration on conformational exchange contributions, $^{15}N$ $R_2$ was measured at 25°C for three different protein concentrations: 94, 470 and 940 μM. No significant change in the $R_2$ values was observed with concentration.

### 2. Analysis of the $^{1}H^{N}$ and $^{15}N$ NMR relaxation dispersion data

The $^{1}H^{N}$ and $^{15}N$ relaxation dispersion data of JIP1-SH3 were analyzed simultaneously for the two magnetic field strengths according to a two-site exchange model using the program ChemEx (https://github.com/gbouvignies/ChemEx). In the first step, the dispersion data of residues showing the largest conformational exchange contributions were analyzed simultaneously. In the second step, the extracted exchange rate constant, $k_{EX}$, and the population of the minor state, $p_{minor}$, were fixed and relaxation dispersion data were included for all residues to extract their chemical shift differences, $\Delta\delta_{CPMG}$, between the major and the minor state.

For the H493A and V517A variants, the $^{15}N$ and $^{1}H^{N}$ relaxation dispersion data allow to extract the exchange rate constant, however, due to their fast exchange behavior, the chemical shift differences, $\Delta\delta_{CPMG}$, remain correlated with the population, $p_{minor}$, of the minor state. We therefore carried out a grid search analyzing the dispersion data for the two variants by varying the population of the minor state from 1% to 20%, in steps of 1%. For each population, a two-step procedure was used as described above for the WT protein. For each fit, the chemical shift differences, $\Delta\delta_{CPMG}$, for the two variants were compared to those extracted for the WT protein. We note that by comparing the $\Delta\delta_{CPMG}$ values, rather than the observable chemical shifts of the WT protein with the two variants, contributions to the experimental chemical shifts from the mutation sites are largely avoided. Optimal agreement between the two chemical shift data sets was obtained for a population of 11% for the V517A variant and of 10% for the H493A variant. As the analysis of the relaxation dispersion data only provide the absolute values of the $\Delta\delta_{CPMG}$ values, we used the observable chemical shifts of the two variants (Fig. 3d, e) to determine which conformation (staggered or eclipsed) corresponds to the minor state in the case of the two variants. The NMR data show that the H493A variant is mainly in an eclipsed conformation (90% - eclipsed, 10% - staggered), while the V517A variant is mainly in a staggered conformation (11% - eclipsed, 89% - staggered) (Fig. 3f).

### 3. Analysis of the $^{13}C$ NMR relaxation dispersion data

The aromatic side chain $^{13}C\varepsilon$ CPMG relaxation dispersion data of Y526 were analyzed simultaneously for the three magnetic field strengths according to a two-site exchange model by ChemEx (Fig. 4b). The analysis yields an exchange rate constant of $k_{EX} = 2400 \pm 300\ s^{-1}$, a population of the minor state of $p_{minor} = 2.9 \pm 0.2$ % with a chemical shift difference of $\Delta\delta_{CPMG}$ = 2.1 ppm. The values of $k_{EX}$ and $p_{minor}$ are in close agreement with those extracted from analysis of the $^{15}N$ and $^{1}H^{N}$ relaxation dispersion data according to a two-site exchange model

($k_{EX}$ = 2600 ± 70 $s^{-1}$ and $p_{minor}$ = 2.8 ± 0.1 %). This shows that the full ring flipping (180°) does not significantly contribute to the observed $^{13}C$ CPMG relaxation dispersion either because the two epsilon protons have degenerate chemical shifts or, more likely, that the ring flipping process is very fast and that a potential exchange contribution is not quenched by the CPMG frequencies probed by the experiment. We cross-validated this result against on-resonance $^{13}C\varepsilon$ $R_{1\rho}$ relaxation dispersion of Y526 by back-calculating the expected $R_{1\rho}$ profile from the values of $k_{EX}$, $p_{minor}$ and $\Delta\delta_{CPMG}$ derived from the $^{13}C\varepsilon$ CPMG relaxation dispersion experiments (Fig. 4c). Excellent agreement between the experiment and theory is obtained, further supporting the conclusion that ring flipping of Y526 is very fast with an estimated exchange rate of $k_{EX}$ > 50000 $s^{-1}$.

## Supplementary Figures

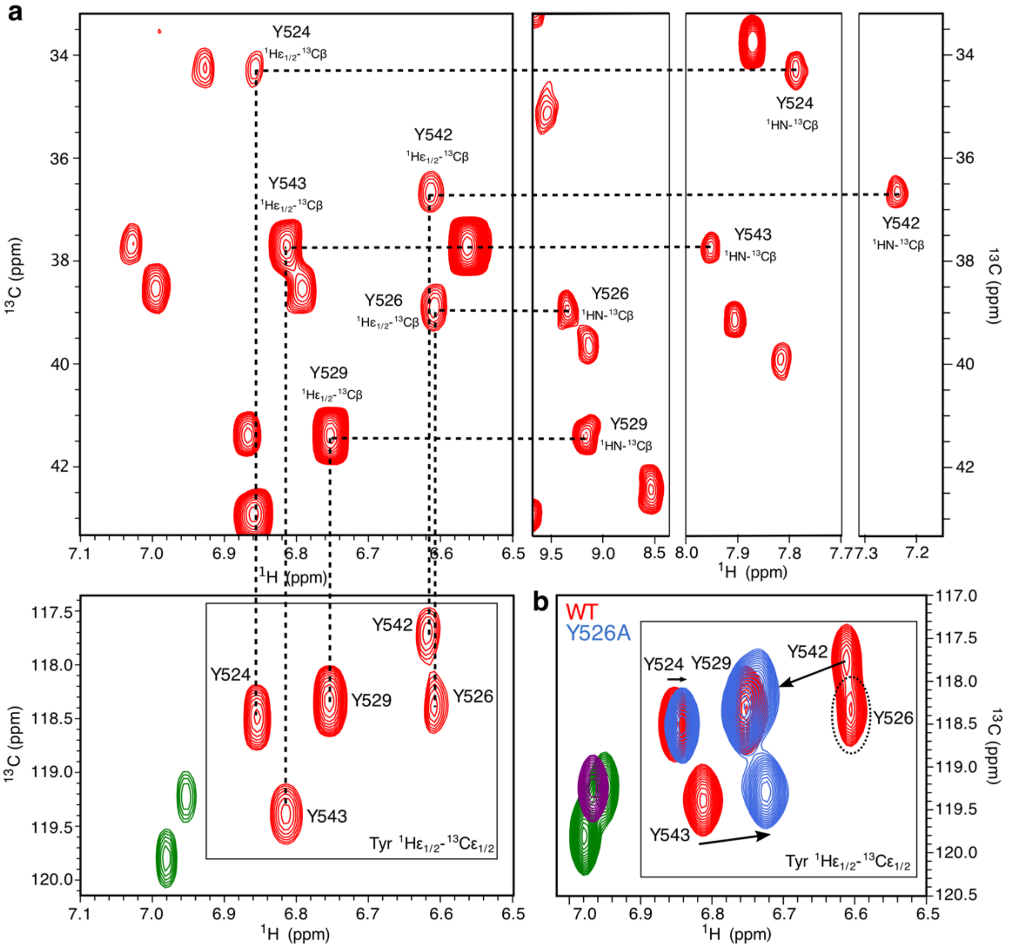


**Supplementary Fig. 1. Assignment of the five tyrosine $^{13}$Cε-$^{1}$Hε resonances of JIP1-SH3 at 45°C. a**, Strips showing the assignment of the tyrosine $^{13}$Cε-$^{1}$Hε correlations. Strips of the intra-residue HNCACB experiment are shown (top right) with dashed lines showing the correlations with the (Hβ)Cβ(CγCδCε)Hε experiment (top left) and with the $^{1}$H-$^{13}$C HSQC experiment (bottom left). The assignment was transferred to 15°C by following the resonances as function of temperature in the $^{1}$H-$^{13}$C HSQC experiment (Extended Data Fig. 10a). **b**, Superposition of the tyrosine $^{13}$Cε-$^{1}$Hε region of the $^{1}$H-$^{13}$C HSQC spectrum of WT JIP1-SH3 (red – positive contours, green – negative contours) and the Y526A variant (blue – positive contours, magenta – negative contours).

## Supplementary Tables

**Supplementary Table 1. Conformational exchange contributions, $R_{EX}$, extracted from $^{15}N$ and $^{1}H^{N}$ CPMG relaxation dispersion data acquired at 15°C of JIP1-SH3 and JIP1-SH3-Y526A.** The exchange contributions were obtained as the difference between $R_{2eff}$ at low (31 Hz for $^{15}N$ and 50 Hz for $^{1}H^{N}$) and high (1 kHz for $^{15}N$ and 2 kHz for $^{1}H^{N}$) CPMG frequencies.

| Residue Number | JIP1-SH3 | | | | JIP1-SH3-Y526A | |
|---|---|---|---|---|---|---|
| | $^{15}N$ $R_{EX}$ ($s^{-1}$) 850 MHz | $^{15}N$ $R_{EX}$ ($s^{-1}$) 600 MHz | $^{1}H$ $R_{EX}$ ($s^{-1}$) 950 MHz | $^{1}H$ $R_{EX}$ ($s^{-1}$) 600 MHz | $^{15}N$ $R_{EX}$ ($s^{-1}$) 700 MHz | $^{1}H$ $R_{EX}$ ($s^{-1}$) 600 MHz |
| **M489** | 0.74 ± 0.71 | 1.44 ± 0.71 | 0.85 ± 1.12 | 1.38 ± 1.38 | - | - |
| **E490** | -0.06 ± 0.71 | 0.29 ± 0.71 | 2.33 ± 0.71 | 2.58 ± 0.71 | 0.29 ± 0.71 | 3.11 ± 0.71 |
| **Q491** | -0.11 ± 0.71 | 0.37 ± 0.71 | 0.39 ± 0.71 | 1.08 ± 0.71 | 0.10 ± 0.71 | -0.13 ± 0.71 |
| **T492** | 0.53 ± 0.71 | 0.32 ± 0.89 | 1.49 ± 2.01 | 1.96 ± 2.78 | 0.25 ± 0.71 | 2.13 ± 0.71 |
| **H493** | -0.15 ± 0.71 | 0.43 ± 0.71 | 1.98 ± 1.15 | 1.08 ± 0.71 | -0.01 ± 0.71 | 0.61 ± 0.71 |
| **R494** | 1.40 ± 0.71 | -0.24 ± 0.71 | 7.87 ± 1.47 | 3.53 ± 1.62 | 1.27 ± 0.71 | 1.37 ± 0.71 |
| **A495** | 0.88 ± 0.71 | 1.35 ± 0.71 | 1.07 ± 1.14 | 2.40 ± 1.51 | 1.36 ± 0.71 | -0.06 ± 0.71 |
| **I496** | 9.03 ± 1.10 | 7.33 ± 1.49 | 43.0 ± 8.2 | 18.3 ± 5.5 | 2.20 ± 0.71 | 2.45 ± 0.71 |
| **F497** | 3.00 ± 0.71 | 0.42 ± 0.77 | 3.41 ± 3.52 | 2.36 ± 2.03 | 2.51 ± 0.71 | 3.82 ± 0.71 |
| **R498** | 6.45 ± 4.74 | 3.51 ± 3.82 | 66.4 ± 106.4 | 27.3 ± 14.0 | 2.19 ± 0.71 | 2.77 ± 0.71 |
| **F499** | 0.67 ± 0.71 | 1.23 ± 0.93 | 21.7 ± 3.2 | 7.44 ± 3.39 | 1.45 ± 0.71 | 0.02 ± 0.71 |
| **V500** | 2.86 ± 1.26 | -0.10 ± 1.77 | 33.0 ± 18.0 | 4.16 ± 7.88 | 1.00 ± 0.71 | 2.13 ± 0.71 |
| **P501** | - | - | - | - | - | - |
| **R502** | 1.23 ± 0.78 | 1.71 ± 1.49 | 7.07 ± 6.32 | -4.44 ± 5.75 | 0.32 ± 0.71 | 2.72 ± 0.71 |
| **H503** | 0.00 ± 0.71 | -0.46 ± 0.71 | 9.47 ± 2.74 | 6.99 ± 2.49 | 0.21 ± 0.71 | 2.67 ± 0.71 |
| **E504** | 0.22 ± 0.71 | 0.26 ± 0.71 | -0.11 ± 1.69 | 1.49 ± 1.70 | -0.02 ± 0.71 | -2.96 ± 0.71 |
| **D505** | 0.10 ± 0.71 | 0.95 ± 0.73 | 29.9 ± 3.2 | 16.1 ± 1.9 | 0.40 ± 0.71 | 1.35 ± 0.71 |
| **E506** | -0.07 ± 0.71 | -0.05 ± 0.71 | 2.57 ± 1.21 | 2.82 ± 1.30 | 0.42 ± 0.71 | -1.71 ± 0.71 |
| **L507** | -0.49 ± 0.71 | 1.65 ± 1.03 | 25.9 ± 5.6 | 15.6 ± 4.2 | 1.13 ± 0.71 | -3.95 ± 0.71 |
| **E508** | 0.31 ± 0.71 | 0.30 ± 0.71 | 0.77 ± 1.11 | 0.29 ± 1.38 | 0.28 ± 0.71 | 0.44 ± 0.71 |
| **L509** | 0.58 ± 1.35 | 2.00 ± 2.09 | 57.1 ± 13.8 | 34.0 ± 9.3 | -0.05 ± 0.71 | 2.55 ± 0.71 |
| **E510** | 3.02 ± 0.71 | 2.22 ± 0.71 | -0.83 ± 1.32 | -1.52 ± 1.76 | 0.60 ± 0.71 | -0.65 ± 0.71 |
| **V511** | 1.05 ± 0.71 | 0.13 ± 0.71 | 0.55 ± 1.23 | 0.77 ± 1.33 | 1.07 ± 0.71 | -0.23 ± 0.71 |
| **D512** | -0.18 ± 0.71 | -0.50 ± 0.72 | -0.54 ± 2.34 | -2.48 ± 2.70 | 0.00 ± 0.71 | 1.39 ± 0.71 |
| **D513** | 0.00 ± 0.71 | 1.18 ± 0.71 | 1.43 ± 0.81 | 1.55 ± 0.93 | 0.91 ± 0.71 | 4.49 ± 0.71 |
| **P514** | - | - | - | - | - | - |
| **L515** | -0.14 ± 0.71 | -0.56 ± 0.71 | -1.13 ± 1.41 | 1.59 ± 1.72 | 0.26 ± 0.71 | 2.86 ± 0.71 |
| **L516** | 1.23 ± 0.71 | 1.22 ± 0.71 | -0.35 ± 1.22 | -1.11 ± 1.56 | 0.78 ± 0.71 | -2.01 ± 0.71 |
| **V517** | 2.55 ± 0.71 | 3.92 ± 0.89 | -1.23 ± 2.11 | -2.65 ± 2.36 | 1.23 ± 0.71 | -1.00 ± 0.71 |
| **E518** | 3.05 ± 0.71 | 3.07 ± 1.13 | 14.4 ± 3.6 | 5.30 ± 3.57 | 1.15 ± 0.71 | 0.43 ± 0.71 |
| **L519** | 21.3 ± 1.2 | 16.6 ± 1.4 | 17.7 ± 2.1 | 7.71 ± 1.48 | 0.38 ± 0.71 | 1.91 ± 0.71 |
| **Q520** | 19.1 ± 3.4 | 17.6 ± 3.1 | 27.2 ± 5.0 | 14.4 ± 3.1 | 0.92 ± 0.71 | 1.27 ± 0.71 |
| **A521** | 24.2 ± 3.2 | 18.5 ± 3.1 | 70.5 ± 15.2 | 25.3 ± 5.1 | 1.08 ± 0.71 | 2.16 ± 0.71 |
| **E522** | 0.62 ± 0.71 | 0.42 ± 0.73 | 24.4 ± 4.0 | 9.23 ± 2.42 | 0.10 ± 0.71 | 3.46 ± 0.71 |
| **D523** | 7.94 ± 4.79 | 4.03 ± 2.98 | 44.1 ± 11.5 | 41.5 ± 5.7 | 0.74 ± 0.71 | 1.38 ± 0.71 |
| **Y524** | 2.88 ± 0.86 | 1.66 ± 1.02 | 35.9 ± 4.5 | 17.5 ± 2.3 | 1.59 ± 0.71 | 2.97 ± 0.71 |
| **W525** | 12.4 ± 1.0 | 10.0 ± 1.2 | 38.4 ± 4.4 | 26.5 ± 2.4 | 0.34 ± 0.71 | 4.86 ± 0.71 |
| **Y526** | 6.01 ± 0.71 | 3.64 ± 0.93 | 19.6 ± 4.2 | 11.3 ± 3.2 | 0.19 ± 0.71 | 1.14 ± 0.71 |
| **E527** | 4.49 ± 0.71 | 5.13 ± 0.71 | 10.4 ± 1.5 | 7.57 ± 1.56 | -0.05 ± 0.71 | 0.42 ± 0.71 |
| **A528** | 1.44 ± 0.71 | 1.65 ± 0.71 | 2.81 ± 1.11 | 2.00 ± 1.22 | 0.79 ± 0.71 | -0.53 ± 0.71 |
| **Y529** | 0.05 ± 0.71 | -0.42 ± 0.71 | 6.63 ± 2.12 | 2.52 ± 2.29 | 0.23 ± 0.71 | 1.13 ± 0.71 |
| **N530** | 0.95 ± 0.71 | 0.58 ± 0.71 | -0.26 ± 1.19 | 0.29 ± 1.45 | 0.90 ± 0.71 | -0.20 ± 0.71 |
| **M531** | -0.08 ± 0.71 | -0.27 ± 0.71 | -1.54 ± 1.80 | -2.23 ± 2.39 | 0.70 ± 0.71 | -2.23 ± 0.71 |
| **R532** | -0.17 ± 0.71 | 0.33 ± 0.71 | 0.95 ± 1.05 | 3.03 ± 1.31 | 0.03 ± 0.71 | -0.67 ± 0.71 |

| | | | | | | |
|---|---|---|---|---|---|---|
| **T533** | 0.95 ± 0.71 | 1.37 ± 0.71 | 1.56 ± 1.04 | -0.56 ± 1.31 | 0.73 ± 0.71 | 1.86 ± 0.71 |
| **G534** | 0.14 ± 0.71 | 0.04 ± 0.71 | 0.22 ± 1.06 | 0.11 ± 1.22 | 0.08 ± 0.71 | 2.17 ± 0.71 |
| **A535** | -0.31 ± 0.71 | 0.26 ± 0.71 | 0.81 ± 0.74 | 0.46 ± 1.01 | 0.50 ± 0.71 | -0.13 ± 0.71 |
| **R536** | -0.45 ± 0.71 | 0.33 ± 0.71 | 1.54 ± 0.71 | 0.59 ± 0.79 | -0.24 ± 0.71 | 0.64 ± 0.71 |
| **G537** | 1.17 ± 0.71 | 1.09 ± 0.71 | 2.28 ± 1.02 | 2.30 ± 1.35 | 0.60 ± 0.71 | 1.59 ± 0.71 |
| **V538** | 1.12 ± 0.71 | 1.23 ± 0.72 | 5.23 ± 2.77 | 0.41 ± 3.03 | 0.48 ± 0.71 | -3.84 ± 0.71 |
| **F539** | 5.82 ± 0.71 | 4.59 ± 0.71 | 4.53 ± 1.34 | 1.79 ± 1.55 | 0.18 ± 0.71 | 1.04 ± 0.71 |
| **P540** | - | - | - | - | - | - |
| **A541** | 10.7 ± 0.7 | 6.65 ± 0.89 | 15.8 ± 2.5 | 4.57 ± 2.24 | -0.02 ± 0.71 | 0.53 ± 0.71 |
| **Y542** | 2.35 ± 1.91 | 4.31 ± 2.42 | 50.2 ± 16.1 | 36.6 ± 10.7 | 1.09 ± 0.71 | 0.31 ± 0.71 |
| **Y543** | 16.8 ± 1.9 | 10.0 ± 1.9 | 39.0 ± 6.0 | 31.3 ± 4.5 | 1.43 ± 0.71 | 2.10 ± 0.71 |
| **A544** | 11.4 ± 0.7 | 7.96 ± 0.73 | 17.8 ± 1.7 | 9.75 ± 1.19 | 0.14 ± 0.71 | 1.91 ± 0.71 |
| **I545** | 0.13 ± 0.71 | 1.07 ± 0.71 | 8.02 ± 1.33 | 6.89 ± 1.50 | 5.67 ± 0.71 | 3.39 ± 0.71 |
| **E546** | 1.46 ± 0.71 | 1.52 ± 0.71 | -0.06 ± 0.82 | -0.25 ± 1.01 | 4.46 ± 0.71 | 2.98 ± 0.71 |
| **V547** | 1.69 ± 0.71 | 2.58 ± 0.71 | 0.10 ± 0.90 | 1.64 ± 1.13 | 3.33 ± 0.71 | 0.70 ± 0.71 |
| **T548** | -0.16 ± 0.71 | 0.10 ± 0.71 | 1.69 ± 0.71 | 1.63 ± 0.71 | 0.48 ± 0.71 | 2.87 ± 0.71 |
| **K549** | 0.10 ± 0.71 | 0.32 ± 0.71 | 1.08 ± 0.71 | 1.90 ± 0.71 | 0.59 ± 0.71 | 2.74 ± 0.71 |
