# Supplementary material for: Visualizing protein breathing motions associated with aromatic ring flipping
Source: Nature. 2022 Feb 16;602(7898):695–700. doi: 10.1038/s41586-022-04417-6 (PMC8866124; doi:10.1038/s41586-022-04417-6)
Supplement: Supplementary file 2 — Reporting Summary [file 41586_2022_4417_MOESM2_ESM.pdf]

nature portfolio

Corresponding author(s): Andres Palencia & Malene Ringkjøbing Jensen

Last updated by author(s): Dec 22, 2021

Reporting Summary

Nature Portfolio wishes to improve the reproducibility of the work that we publish. This form provides structure for consistency and transparency in reporting. For further information on Nature Portfolio policies, see our Editorial Policies and the Editorial Policy Checklist.

Statistics

For all statistical analyses, confirm that the following items are present in the figure legend, table legend, main text, or Methods section.

n/a	Confirmed	
☐	☒	The exact sample size (*n*) for each experimental group/condition, given as a discrete number and unit of measurement
☒	☐	A statement on whether measurements were taken from distinct samples or whether the same sample was measured repeatedly
☒	☐	The statistical test(s) used AND whether they are one- or two-sided<br>*Only common tests should be described solely by name; describe more complex techniques in the Methods section.*
☒	☐	A description of all covariates tested
☐	☒	A description of any assumptions or corrections, such as tests of normality and adjustment for multiple comparisons
☐	☒	A full description of the statistical parameters including central tendency (e.g. means) or other basic estimates (e.g. regression coefficient) AND variation (e.g. standard deviation) or associated estimates of uncertainty (e.g. confidence intervals)
☒	☐	For null hypothesis testing, the test statistic (e.g. *F*, *t*, *r*) with confidence intervals, effect sizes, degrees of freedom and *P* value noted<br>*Give P values as exact values whenever suitable.*
☒	☐	For Bayesian analysis, information on the choice of priors and Markov chain Monte Carlo settings
☒	☐	For hierarchical and complex designs, identification of the appropriate level for tests and full reporting of outcomes
☒	☐	Estimates of effect sizes (e.g. Cohen's *d*, Pearson's *r*), indicating how they were calculated

Our web collection on statistics for biologists contains articles on many of the points above.

Software and code

Policy information about availability of computer code

Data collection

NMR data acquisition: TopSpin version 3.5, VnmrJ version 3.1
X-ray data collection: MXCube version 3

Data analysis

Analysis of NMR relaxation dispersion data: Open source software ChemEx version 2021.4.0-dev2 (https://github.com/gbouvignies/ChemEx)
Pocket volume calculations: POVME version 3.0
Indexing and integration, X-ray crystallography data: XDS version 20200417, autoProc version 1.1.7
Data reduction, X-ray crystallography: Pointless version 1.12.2, Aimless version 0.7.4
Molecular replacement, X-ray crystallography: Phaser version 2.8.3
Model building and refinement, X-ray crystallography: Coot version 0.8.9.2, Refmac version 5.8.0258, CCP4I version 7.0.078 or 7.1.008

For manuscripts utilizing custom algorithms or software that are central to the research but not yet described in published literature, software must be made available to editors and reviewers. We strongly encourage code deposition in a community repository (e.g. GitHub). See the Nature Portfolio guidelines for submitting code & software for further information.

Data

Policy information about availability of data

All manuscripts must include a data availability statement. This statement should provide the following information, where applicable:

- Accession codes, unique identifiers, or web links for publicly available datasets
- A description of any restrictions on data availability
- For clinical datasets or third party data, please ensure that the statement adheres to our policy

Protein structure data have been deposited in the PDB database with accession codes: 7NYK (JIP1- SH3), 7NZB (JIP1-SH3-V517L), 7NYO (JIP1-SH3-A541L), 7NYL (JIP1-

SH3-H493A), 7NYM (JIP1-SH3-V517A), 7NZC (POSH-SH3-1) and 7NZD (POSH-SH3-4). The 1H, 13C and 15N chemical shifts of JIP1-SH3 have been deposited in the Biological Magnetic Resonance Data Bank with accession codes: 50814 (JIP1-SH3), 50817 (JIP1-SH3-Y526A), 50816 (JIP1-SH3-V517A), 50818 (JIP1-SH3-H493A), 50815 (JIP1-SH3-A541L).
SH3 domain structures for molecular replacement were retrieved from the PDB with accession codes: 2FPE (JIP1), 2LJ1 (Sorbin and SH3 domain-containing protein 1) and 2SRC (tyrosine protein kinase C-Src). Structures for the proteome-wide SH3 sequence analysis were retrieved from the PDB with accession codes: 1CSK, 3A98, 2O9S, 5VEI and 4LNP.

Field-specific reporting

Please select the one below that is the best fit for your research. If you are not sure, read the appropriate sections before making your selection.

☒ Life sciences ☐ Behavioural & social sciences ☐ Ecological, evolutionary & environmental sciences

For a reference copy of the document with all sections, see nature.com/documents/nr-reporting-summary-flat.pdf

Life sciences study design

All studies must disclose on these points even when the disclosure is negative.

Sample size	Statistical methods were not used to determine sample size.
Data exclusions	Data were not excluded.
Replication	Each crystal structure was solved from a single crystal. All nmr dispersion experiments were performed once with technical replicates as described in the Methods section.
Randomization	There was no randomized sample allocation in this work. All tested protein designs received identical treatment.
Blinding	Blinding is not relevant for structure determination by X-ray crystallography, blinding was not necessary for other methods used in this study.

Reporting for specific materials, systems and methods

We require information from authors about some types of materials, experimental systems and methods used in many studies. Here, indicate whether each material, system or method listed is relevant to your study. If you are not sure if a list item applies to your research, read the appropriate section before selecting a response.

Materials & experimental systems

n/a	Involved in the study	
☒	☐	Antibodies
☒	☐	Eukaryotic cell lines
☒	☐	Palaeontology and archaeology
☒	☐	Animals and other organisms
☒	☐	Human research participants
☒	☐	Clinical data
☒	☐	Dual use research of concern

Methods

n/a	Involved in the study	
☒	☐	ChIP-seq
☒	☐	Flow cytometry
☒	☐	MRI-based neuroimaging
